# Tax evasion and government size: evidence from Italian provinces

**DOI:** 10.1007/s40888-021-00227-7

**Published:** 2021-04-02

**Authors:** Elena D’Agostino, Marco Alberto De Benedetto, Giuseppe Sobbrio

**Affiliations:** 1 Department of Economics, University of Messina, Via dei Verdi, 75, 98122 Messina, Italy; 2 Department of Economics, Statistics and Finance, University of Calabria, Via Ponte Bucci, 87036 Arcavacata di Rende, CS Italy

**Keywords:** Tax evasion, Public spending, Public goods, GMM, Efficiency, C33, C59, H20, H21, H26, H50

## Abstract

We analyze the impact of government size, measured by total spending per capita, on tax evasion at the provincial level in Italy over the period 2001–2015. In order to solve endogeneity issues we rely on a system GMM and find that public expenditure negatively affects tax evasion, as taxpayers perceive the government is efficiently spending resources coming from the tax levy. Results are confirmed when we (1) consider expenditures related to long-term investments, namely capital spending per capita, and (2) directly test the impact of government efficiency on tax evasion. In addition, we show that the impact of public spending is heterogeneous across geographical areas: an increase in public expenditure leads to a downward shift in tax evasion only in the northern part of Italy, characterized by a relatively larger initial level of public goods provision.

## Introduction

In Italy tax evasion represents a big issue for the national economy and public finance. The losses due to tax evasion are estimated to be 110 billion euros annually up to 2015 (Relazione fiscale e contributiva 2015), where the widest opportunities to cheat fiscal authorities are available to entrepreneurs and professionals. In fact, 56.3% of them is estimated to pay no taxes or less taxes than the amount due (Evasione fiscale 2015).

Moreover, a dramatic picture of the Italian situation emerges overtime from the Italian Statistics Institute (ISTAT) and the Italian Revenue Agency (Agenzia delle Entrate, IRA henceforth) data sources: in 1981 tax evasion in Italy was about 28 billion euros, equivalent to 7–8% of GDP, and in 2015 this share has risen to between 16.3 and 17.5% of GDP, for a total that fluctuates between 255 and 275 billion euros of taxable income subtracted from the tax authorities, with strong repercussions on the public deficit and the consequent public debt, placing Italy on top of the European and OECD countries for tax evasion. Nonetheless, tax evasion seems to be heterogeneous among regions: in northern Italy, where the most significant share of business and income is realized, more taxable income is evaded in absolute monetary value, while the south has the record for number of evaders.

The public finance literature has mainly focused, on the one hand, on the determinants of tax evasion, such as tax morale, tax burden, quality of institutions, density of regulation and unemployment rate (see among others, Torgler and Schneider 2007; Schneider and Enste 2002; Schneider et al. 2010), and on the other hand, on the relationship between corruption and the shadow economy (Johnson et al. 1997; Dreher and Schneider 2010), whereas little is known on the impact of public spending on taxpayers’ behavior.

In particular, as stated by Frye and Shleifer (1997), government might be perceived as operating as a ‘grabbing hand’ or a ‘helping hand’. In the first case, an increase in government size leads to state predation and to an upward shift in tax evasion. Conversely, if the ‘helping hand’ role dominates, an increase in the size of government would strengthen its state capacity, reducing in turn tax evasion. Whether a ‘grabbing hand’ or a ‘helping hand’ dominates depends on the quality of government and of public goods provided. In this perspective, Li and Ma (2015) estimate the impact of county government size on the relationship between firms’ reported profit and imputed profit based on the national income accounts over the period 1998–2005 in China. A larger government is positively correlated with more severe tax evasion, and this effect is stronger when local governance becomes worse.

The goal of our paper is to shed light on how government size, as measured by public spending, affects tax evasion using data at the provincial level in Italy over the period 2001–2015 that are not publicly available and provided by the Italian Revenue Agency. Findings show that an increase in government size leads to a downward shift in tax evasion, since taxpayers perceive the government is efficiently managing the public “ pursue” , leading in turn to a general gratification of taxpayers. This is essentially true when in the face of an increase in public expenses, taxpayers do feel satisfied about the quality of public goods provided by provincial governments.

Findings are not affected by the inclusion of covariates, such as population size, unemployment rate, percentage of elderly people and GDP per capita, considered by the mainstream literature as potential determinants of taxpayers’ behavior. In addition, we take into account in our analysis that the increase in government size might be due to inefficiencies produced by bureaucrats and focus in turn on those expenses that are more likely to be under direct control of office-holders, i.e. current expenditures per capita. Results show that current spending does not affect the propensity to evade taxes. Conversely, an increase in capital expenditure that should be the type of spending less under the direct control of local policymakers/bureaucrats leads to a decrease in tax evasion by 3.8%. These results are confirmed when we directly test for the impact of government efficiency on tax evasion, highlighting that the more the efficiency is the higher is the “ cooperative” reaction of taxpayers in terms of a better attitude towards fiscal duties.

Finally, Italian provinces are quite heterogeneous in terms of social capital and income with the south that is poor and endowed with a lower level of social capital compared to the north. Given that tax evasion (public spending) is higher (lower) in the southern part of Italy, we test if public spending differently affects taxpayers’ behavior in these two main geographical areas. Findings show a negative government size effect in those regions where public expenditure is historically higher: a conclusion that finds theoretical support in Falkinger (1988) and Cowell and Gordon (1988), and reconsiders the role of morale, intrinsic motivations and reputational elements in explaining changes in taxpayers’ behavior.

We contribute to the existing literature in different ways. First, we directly test the effect of public expenditures on actual tax evasion, using a confidential and unique data set provided by the IRA, rather than on tax morale, as in Barone and Mocetti (2011).^1^ Moreover, compared to what Li and Ma (2015) present, we focus not only on taxes evaded by companies but on the overall tax gap and show that our effect is driven by an increase in capital expenditures and is at play only in areas characterized by a low level of complaints by citizens, and in those areas where the intervention of provincial governments is stronger, suggesting that citizens’ perception about the quality of public goods provided is an important driver mitigating the impact of public spending on tax evasion and that government is rated by taxpayers as ‘helping hand’ in Italy.

Second, related to the previous point, we exploit the panel structure of our data set and hinge on a system-GMM technique to recover the impact of public spending on tax evasion, solving potential endogeneity issues, such as the omitted variable bias and the measurement error affecting the variable of interest. It is worth to stress that since it is quite hard to envisage an appropriate and clean instrumental variable approach to instrument government spending, we employ a GMM framework which generates instruments internally. Moreover, while we take the direction of causality from government expenditures to tax evasion for granted, we are aware that our findings might be affected by reverse causality. In this regard, it is also important to state that the empirical methodology used in our investigation does not establish causality. Therefore, our results should be only seen as conditional correlations. However, such correlations are interesting because they are statistically robust, and in turn our results suggest that theories analyzing the relationship between government spending and/or efficiency and tax evasion should be consistent with such correlations. Nonetheless, we do also present, as further robustness checks, (1) a Granger test on the direction of causality between our main variable of interest and tax evasion proposing that causality runs from the former to the latter, and (2) an IV approach in which we use the historical level of spending per capita borne by province governments in 1998 as an instrument for government size over the period 2007–2015, reaching similar results.

Last but not least, we further go deep into some channels through which public spending might impact tax evasion. In fact, (1) we exploit geographical heterogeneity among Italian provinces, and (2) we show that areas of public interventions which are in close connection with the everyday needs of many citizens do impact taxpayers’ behaviour, whereas narrow sectors of expenditures, which instead target specific groups of taxpayers, are ineffective.

We also propose in the Appendix a simple two-period model in which in every period a government sets a policy consisting in the tax rate and in the level of public expenditure. If expenditure is set low, the government provides an essential public good, and in the opposite case it can decide (1) to invest the extra-expenditure in a public service of either general or specific interest for taxpayers (good quality) or (2) to finance patronage and bureaucracy (bad quality), where the latter strategy is less costly than the former. Taxpayers decide whether to pay taxes after they observe the tax rate and the level of public expenditure, but not its quality in the first period. We assume that taxpayers prefer (and are therefore willing to pay more for) high-quality rather than low-quality public goods; moreover, we assume that all taxapayers care more about a public good of general interest (e.g. transportation) and only some of them equally care of a public good of specific interest (e.g. education). In order to verify how taxpayers’ satisfaction influences the decision to evade taxes, we assume that they are motivated by the return they get from public expenditure. Precisely, holding a priori beliefs in favor or against the government in period 1 (accordingly, we distinguish between optimistic and pessimistic taxpayers), some taxpayers become aware of the quality at the end of period 1 and, if not satisfied by the policy implemented, decide to punish the government by evading taxes in period 2 when the quality cannot be changed. The model predicts that in equilibrium tax evasion increases (viz. decreases) if high public expenditure of bad quality (viz. of good quality and of general interest) is provided, whereas the behavior of taxpayers is ambiguous if high public expenditure of good quality and of specific interest is provided in period 1.

The paper is structured as follows. In Sect. 2 we discuss the main literature on the determinants of tax evasion. In Sect. 3 we describe our sample and in Sect. 4 we present the methodology applied in our empirical exercise and the preliminary results. Then, Sect. 5 shows results taking into account potential endogeneity issues. Section 6 highlights some heterogeneities of our findings, whereas Sect. 7 presents some robustness checks. Section 8 concludes.

## Literature review

In the seminal work by Alligham and Sandmo (1972) the individual decision of evading taxes is modelled as a gamble, and tax evasion is found to be negatively correlated to penalty and the probability of detection. This approach has been criticized as it is non-satisfactory in explaining the tax evasion phenomenon and its evidence all around the world. More generally, the criticism involves the paradigm of the traditional homo oeconomicus as a rational selfish decision maker (Andreoni et al. 1998; Slemrod 2007). The main new frontier is rather to consider tax evasion as the final decision of a much more complex iter involving individual intrinsic motivations and morale, along with purely economic incentives.

Since the pioneer work by Jackson and Milliron (1986) many key-variables responsible for tax evasion have been evaluated in the literature. Among those related to taxpayers’ characteristics, great relevance has been given to age, gender, education, occupational status and income. In particular, US data from the Taxpayer Compliance Measurement Program show that age negatively affects tax evasion, and people aged 65 or above are less likely to evade taxes (Andreoni et al. 1998): a result confirmed by experimental studies (Baldry 1987; Friedland et al. 1978), and holding true also in those works where age is only used as control variable (Clotfelter 1983; Feinstein 1991). In addition, Tittle (1980) explains the relationship between age and tax evasion pointing out that young people are usually risk-lovers and less sensitive to the risk of penalties.

Education is another important determinant of tax evasion. Jackson and Milliron (1986) argue that education has two potential opposite effects on tax evasion. The better knowledge of the tax system, on the one hand, should favour positive feelings about taxation, turning out in a lower level of tax evasion, but on the other hand, it may also increase the capability of how to evade taxes, leading to a high level of tax evasion. The literature on this topic suggests that the first effect dominates the latter, so that education of taxpayers and tax evasion are negatively correlated (Song and Yarbrough 1978; Wallschutzky 1984; Witte and Woodbury 1985).

Also the unemployment rate has been investigated as a potential factor affecting taxpayers’ behaviour: as unemployed citizens do not gain any salary and are not supposed to pay taxes, the propensity to evade should decrease. Conversely, there are studies focusing on the opposite relationship that is whether tax evasion influences unemployment. Isachsen and Strøm (1981), find that for workers who are able to distinguish between the official and the hidden labour market, an increase in the probability of being caught in tax evasion has a positive impact on their choice toward the official market. Generally speaking, a decline in the labor participation force rate may be associated to a switch of workers from the official market to the hidden market, so that unemployment should generate tax evasion (Contini 1982).

A second strand of the literature has rather focused on contextual and public factors, such as marginal tax rates, sanctions and probability of detection, quality of institutions (Dreher et al. 2009), corruption (Dreher and Schneider 2010; Johnson et al. 1997; Hibbs and Piculescu 2005), and economic freedom (Riahi-Belkaoiu 2004; Richardson 2006) in order to explain the tax evasion phenomenon. In fact, as far as the relationship between tax evasion and tax rate is concerned, Clotfelter (1983) proposes an empirical analysis for the United States, and using cross-sectional data and a Tobit model finds a positive relationship between tax evasion and both marginal tax rate and after-tax income. However, tax rate and income are positively correlated. Feinstein (1991) sorts out this issue exploiting an exogenous change in the tax rate for given levels of income in the United States in both 1982 and 1985: no statistically significant effect of income on tax evasion is detected, although the impact of the marginal tax rate interestingly turns out to be negative and significant. Moving to the psychological determinants of tax evasion, i.e. those related to individual attitudes and behaviour, the literature becomes huge as it has embraced almost every aspect of the so-called tax morale, although fairness, reciprocity and guilt have also been fully explored. For instance, Bordignon (1993) proves that citizens’ intrinsic motivation to pay taxes decreases when the neighbours are more willing to evade. The closest literature to our project is certainly that focusing on tax morale to be intended as the degree of satisfaction taxpayers show to government policies on the provision of public goods and, more generally, on public expenditure. The experimental literature converges to assess that taxpayers are more likely to evade if they feel their money is not well spent (Alm et al. 1992; Webley et al. 1991).

At this aim, Barone and Mocetti (2011) propose an empirical work to analyse the effect of local efficiency on tax morale for Italian municipalities, where tax morale is measured by opinions on public spirit and taxation collected through the 2004 survey that is conducted every two years by the Bank of Italy and involving around 8000 households. Empirical results show that public spending inefficiencies negatively affect citizens’ tax morale and this effect is larger if the level of public spending is lower. Related to this point, Cowell and Gordon (1988) highlight the impact of redistribution through the provision of public goods on the perceived fairness and legitimacy of governments in terms of taxes collection. Their theoretical model predicts a positive relation between tax rate (associated to public expenditure) and tax evasion when public goods are underprovided (because, e.g., the initial tax rate is low); conversely, a further increase in the tax rate once public goods are over-provided would lead to a decrease in tax evasion. Also, authors rise concerns about their own results as intuitively individuals should be more prone to evade taxes when they feel that the government does not use their money properly, especially in the case of over-provision (and not under-provision) of public goods.

Our paper is also related to the huge literature investigating the determinants of government size and their effect on economic outcomes. Above all, there are two main approaches in the literature that study government size and its influence on the growth and development of a country (see for instance, Cerniglia et al. (2017)). The first approach focuses on the market side, i.e. the demand for public services and the changes on the supply side due to new technologies and globalization. In particular, Wagner (1883) law establishes that government size increases due to an increase of demand for public services (security, public order, justice, etc.). On the same line, Bator (1958) proposes market failures as an explanation for the increase of the government activities and public expenditure, whereas Samuelson (1954) focuses on the provision of public goods. On the supply side, Kau and Rubin (1981) identify changes in technology, an increase of market production and an increase in female labour participation as the main drivers of the growth of public expenditures.

The second approach looks at the structure of the state and other local governments, so that political institutions are identified to play a crucial role. On this line, a special focus has been devoted to bureaucracy (Niskanen 1971), interest groups (Stigler 1971), electoral rules (Persson and Tabellini 1999) and fiscal illusions. Related to this point, Alesina and Perotti (1996) note that a complex tax legislation makes it very hard for citizens to understand the real tax pressure and to compare it with the provision of public services. As a result, government size increases due to higher tax revenues and public expenditure.

Motivated by these results, the present study adds to the literature that investigates the effect of government size on the growth and development of the economy estimating a robust impact of public spending, as measured by total, current and capital current expenditures, on the propensity to evade from the payment of taxes at the province level in Italy. In fact, an increase in public spending may reflect on less tax evasion if people realize that they are not victims of this illusive process, and when governments efficiently use money coming from the tax levy. Our work also relates to Li and Ma (2015) findings for China that highlight how a bigger government size is correlated with more severe tax evasion by firms, leading to the conclusion that tax evasion could be the effect of collusion between firms and local governments.

## Data description and sample selection

In our analysis we have adopted different sources of data, and Table 1 reports some descriptive statistics for the main variables used in the empirical exercise. First, to build up our outcome variable, i.e. the tax gap (the propensity to evade from the payment of Value-added tax, IRAP, IRES and IRPEF taxes^2^) we have used unique information on tax evasion at province level provided by the Italian Revenue Agency (Agenzia delle Entrate) over the period 2001–2015.

The common approach to calculate the tax gap (so-called top-down) is based on a comparison between fiscal data and a corresponding macroeconomic aggregate (generally represented by national accounting flows), which incorporates an estimate of the shadow economy, appropriately selected in order to construct an all-encompassing theoretical tax base, that is compared then to the tax base declared by the universe of taxpayers. In international best practices, the top-down method is especially applied in quantifying the tax gap of indirect taxes (VAT, excise duties, etc.). In Italy, however, the presence of a tax on the value of net production, such as IRAP, makes it possible to measure also the tax gap of direct taxes through a top-down approach.^3^

**Table 1:** Descriptive statistics

Variable	Obs	Mean	Std. dev.	Min	Max
Tax gap	1589	0.4213	0.1304	0.1638	0.7761
Total expenditures per capita (ln)	1589	2.8437	0.7494	1.2621	5.7182
Current expenditures per capita (ln)	1589	2.4832	0.7225	0.9840	5.2539
Capital expenditures per capita (ln)	1589	0.7519	0.5508	0.0443	3.2575
Surplus	1589	7.5458	1.2374	0.9545	15.2643
Speed payment	1589	1.1201	0.2493	0.6973	4.1562
Current transfers per capita (ln)	1589	1.3559	0.7964	0.0472	4.7587
Capital transfers per capita (ln)	1589	0.3939	0.3775	0.0042	3.4433
GDP per capita	1565	23,486.51	6,187.70	11,900	51,600
Pop/1000	1589	549.1793	578.1351	86.828	4,342.046
% Elderly people	1589	0.2127	0.0323	0.167	0.3590
Employment rate	1562	0.5765	0.0937	0.3517	0.7269
South	1589	0.3486	0.4766	0	1

Source: Ministry of Internal Affairs, ISTAT and Agenzia delle Entrate

Second, we exploit public expenditures data coming from the municipal balance sheets available on Ministry of Internal Affairs (Ministero degli Interni) website from 1998 to 2015. The budget is the main instrument used to plan the economic and financial management of local governments in which all of information on total inflows and outflows (Entrate e Spese Totali) can be found. Total inflows are essentially divided into current inflows (Entrate Correnti), including tax revenues (Entrate Tributarie), non-tax revenues (Entrate Extra-Tributarie) and transfers (Entrate per Trasferimenti) and capital inflows (Entrate in Conto Capitale), including transfers for investment projects (Trasferimenti di Fondi per Investimenti) and mortgages (Spese per Rimborso Mutui). Current inflows are usually used to finance current outflows (Spese Correnti), including expenses borne for a day-to-day regions’ management, whereas capital outflows (Spese in Conto Capitale) are usually financed through capital inflows.

Since we observe the tax gap for Italian provinces and budget data are available only at the local level in Italy, we aggregate the municipal budget variables at the level of provinces. There are 110 provinces in Italy and they are the intermediate level of local government between the municipalities and regions. Regions are composed of a certain number of provinces which, in turn, are made up of a certain number of municipalities. This implies that each province belongs to one and only one region. After grouping the data at the provincial level, we end up with a panel of 1589 observations, over 15 years (2001–2015) and across 106 provinces.^4^

As highlighted in Table 1, we build our main variables of interest, measuring province government size, as total spending (not including interests) per capita (in logarithm) with a mean of 2.85 and a standard deviation of 0.75, current expenditures per capita and capital expenses per capita with a mean of 2.46 and 0.75 respectively.

**Fig. 1:**
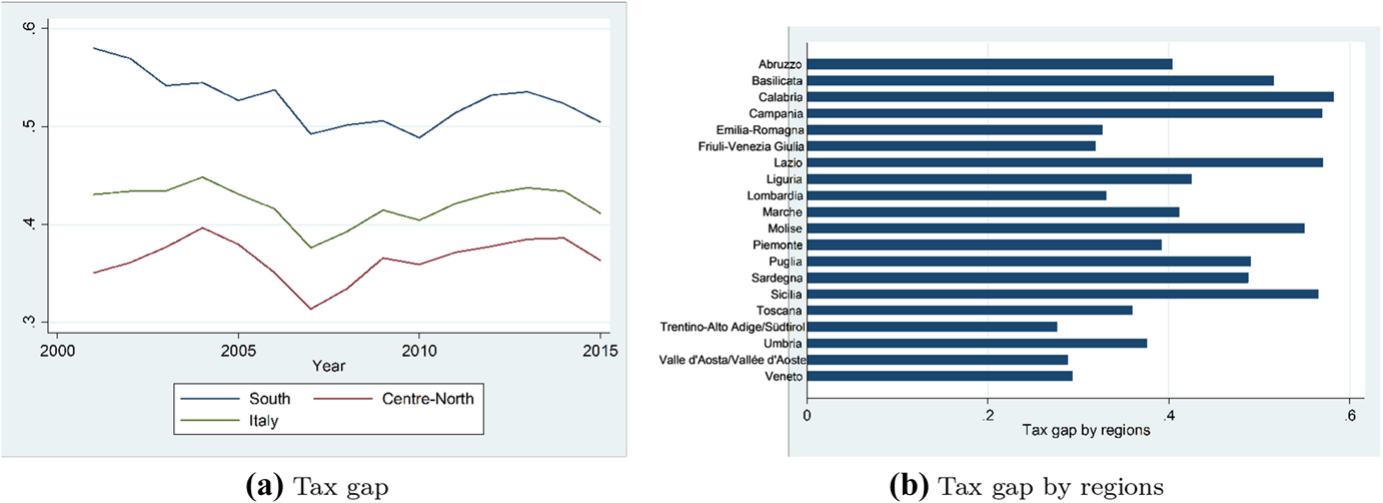
Tax gap per capita over time and by regions

Moreover, in Fig. 1 we plot the pattern of the average tax gap (panel a) to give an insight on how it has changed over the period under scrutiny. The x axis of the graph shows the years while the average tax gap appears on the y axis. In particular, the green line refers to tax evasion for all provinces, whereas the blue and the red line refer to the tax gap in the south and in the north of Italy respectively. Overall, it can be noticed that tax evasion is on average always above 30% and sharply upturns since 2007, when the financial crisis started. Furthermore, regions located in the southern part of Italy^5^ are characterized by a high level of tax gap compared to those in the center-north. For sake of completeness, in panel (b) of Fig. 1 we present the average level of tax evasion by regions.

Finally, in order to control for province demographic characteristics that are largely considered in the literature as main drivers of tax evasion, we exploit information provided by ISTAT on: the size of resident population/1000 (with a mean of 549.18), the average employment rate (with a mean of 0.57), the proportion of people aged 65 or over (with a mean of 0.21), and the GDP per capita at current price (with a mean of 23,486 euros), as a proxy for the province income.

## Methodology and preliminary results

We first estimate a dynamic OLS model with fixed effect at the province level, to study the sign and the magnitude of the correlation between government size and tax evasion, as follows:

1 $$\begin{aligned} \begin{aligned} Tax\,\, Evasion_{it}&=\beta _{0}+\beta _{1}Tax\,\,Evasion_{it-1}+\beta _{2}Public\,\,Expenditures_{it} \\&\quad +X_{it}+\lambda _{i}+\mu _{t}+\varepsilon _{it}, \end{aligned} \end{aligned}$$

where the outcome variable is measured by the tax gap in province *i* at time *t*; $TaxEvasion_{(it-1)}$ is the level of tax gap registered in province *i* at time $t-1$; $PublicExpenditures_{it}$ is measured by the level of spending per capita at the provincial level; $X_{it}$ is a vector containing the potential determinants of tax evasion, such as the unemployment rate, the proportion of elderly people, the number of inhabitants, and the GDP per capita. $\lambda _{i}$ and $\mu _{t}$ are fixed effects at province level and year dummies respectively. The fixed effects $\lambda _{i}$ account for time-invariant characteristics of the province, either observable or unobservable, whereas $\mu _{t}$ measures time-specific common shocks, such as the economic business cycle, affecting all provinces in Italy in a similar way. $\varepsilon _{it}$ is the stochastic error in the model.

Table 2 reports OLS results. In odd columns we only control for province-year dummies, whereas in even columns we add the full set of determinants of tax gap as control variables. Furthermore, in the first two columns the main variable of interest is measured by the level of total expenditures per capita, whereas in the last four specifications we focus on current (columns 3 and 4) and capital (columns 5 and 6) expenditures per capita respectively. In each specification standard errors are robust to heteroskedasticity and clustered at provincial level.

**Table 2:** Tax evasion and spending

Variables	Tax evasion (1)	Tax evasion (2)	Tax evasion (3)	Tax evasion (4)	Tax evasion (5)	Tax evasion (6)
Tax evasion ($t-1$)	0.614*** (0.021)	0.602*** (0.021)	0.617*** (0.022)	0.606*** (0.022)	0.609*** (0.021)	0.597*** (0.021)
Total expenditures	− 0.0153*** (0.0057)	− 0.0159*** (0.0055)				
Current expenditures			− 0.0145* (0.0084)	− 0.0142 (0.0087)		
Capital expenditures					− 0.0078*** (0.0026)	− 0.0079*** (0.0025)
GDP per capita		− 0.0013*** (0.0004)		− 0.0011** (0.0005)		− 0.0014*** (0.0004)
Population/1000		− 0.0010 (0.0010)		0.0010 (0.0010)		− 0.0010 (0.0010)
%Elderly people		− 0.1111 (0.0795)		− 0.0943 (0.0811)		− 0.1040 (0.0819)
Employment rate		− 0.0007 (0.0005)		− 0.0008 (0.0005)		− 0.0007 (0.0005)
Constant	0.229*** (0.226)	0.241*** (0.0336)	0.220*** (0.0299)	0.216*** (0.0356)	0.180*** (0.0101)	0.190*** (0.0293)
Province FE	Yes	Yes	Yes	Yes	Yes	Yes
Year dummies	Yes	Yes	Yes	Yes	Yes	Yes
Observations	1483	1452	1483	1452	1483	1452
R-squared	0.963	0.963	0.963	0.963	0.963	0.963
Number of provinces	106	106	106	106	106	106

OLS with FE results

OLS estimates. We focus on the period 2001–2015. In each specification we control for year dummies and province fixed effects. Standard errors are robust to heteroscedasticity and are clustered at the provincial level (shown in brackets). Significance at the 10% level is represented by *, at the 5% level by **, and at the 1% level by ***

Findings highlighted in the specification reported in column (1) show how the level of total spending per capita is negatively correlated to tax evasion: increasing the main variable of interest by 1% leads to a downward shift in the tax evasion by 1.53% roughly. The main explanation driving our results is that an increase in public expenditure, used as a valid proxy of provincial government size, is well perceived by citizens, as the government is efficiently spending resources coming from the tax levy, leading in turn to a general satisfaction of taxpayers. This generally happens if there is an adequate supply of public goods behind the increase in public expenses, or if the quality of public goods supplied tends to be high. Again our results are consistent with those found in the theoretical literature by which taxpayers are less likely to evade if they feel that their money is well spent (Alm et al. 1992; Webley et al. 1991).

In column (2) our findings remain substantially unchanged when we control for some provinces’ characteristics. Again a 1% increase in the level of total spending per capita reduces the tax gap by 1.59%. As far as the control variables are concerned, we find a negative correlation between the GDP per capita and the propensity to evade taxes that is in line with results highlighted in the literature (see Jackson and Milliron 1986; Song and Yarbrough 1978; Wallschutzky 1984; Witte and Woodbury 1985). Finally, all the other potential determinants of tax evasion (unemployment rate, the proportion of people aged 65 or above, and the population size) do not have any statistically significant impact on our outcome variable, even though they have the expected sign.

In the remaining columns we decompose the overall expenditures into current and capital spending and estimate the effect of each component on tax evasion. In particular, we rely on the public finance literature (see for instance Niskanen 1971) that has tried to explain the different motivations behind the growth of public spending, bringing them back essentially to the state-over-citizen theories of government growth. Here, the size of government is independent from citizen demand and government grows, not because there is a bigger provision of public goods, but because of inherent inefficiencies in public sector activities and incentives facing government bureaucrats.

At this aim, we measure the province government size by expenses that are more directly under control of bureaucrats, i.e. current expenditures per capita (as mentioned before they refer to expenses borne for a day-to-day management of provinces) including, among others, operating expenditures and retributions paid to public employees.

Results displayed in column (4) of Table 2 where we control for province-year dummies and we add the full set of controls as described before, the level of current spending per capita does not produce any significant impact on taxpayers’ propensity to evade taxes. Conversely, as expected, when the focus is on capital expenditures (see column 5 and 6), i.e. those expenses intended to create future benefits, such as infrastructure investment in transport (roads, rail airports), health (water collection and distribution, sewage systems), communication and research spending, we find a negative and statistically significant impact of government size on tax evasion: the more the local governments invest in long-term projects the lower is the perception of taxpayers that money is not well-spent.

We are aware that tax evasion and the level of spending are co-determined, and a simple OLS analysis with fixed effects at province level is not enough to solve any potential issues related both to omitted variable bias (all those time-varying unobserved characteristics at provincial level correlated to the public expenditures that might also affect our outcome variable), and measurement error in the variable of interest. For this reason, in order to recover a robust effect of government size on tax evasion and to solve any potential endogeneity problems, in the next section we present robust results by means of a System Generalized Method of Moments (GMM).

## Endogeneity issues and GMM techniques

### Main results

As previously stated, the OLS results with provincial fixed effects, although robust to heteroskedasticity and with standard errors clustered at province level, are still subject to different types of bias. Particularly relevant in our empirical context is: (1) the dynamic panel bias due to the presence of the lagged dependent variable among the regressors, and (2) the endogeneity bias caused by the correlation between the variable of interest and the error term.

As regards the first issue, in the previous section the positive correlation between the lagged value of tax evasion and the error term of the regression is likely to attenuate the coefficient estimates on the dependent variables. Conversely, in the latter case the potential endogeneity bias may shift the estimated coefficients either upward or downward. In order to avoid both types of bias, a common approach in the literature is to use the Generalized Methods of Moments (GMM estimation) techniques proposed by Arellano and Bond (1991) and Blundell and Bond (1998), which rely on using lagged values of the potentially endogenous covariates (included tax evasion) as instruments. One crucial difference between the two approaches relates to the exact choice of instruments. Arellano and Bond (1991) suggest the use of lags of the endogenous regressors in levels to estimate the specification of interest in first differences. On the other hand, Blundell and Bond (1998) suggest the joint estimation of the specification of interest in levels and in first differences using lags of the endogenous regressors in terms of both levels and first differences.

In our case, we prefer to recover the impact of government size on tax evasion by means of a one-step system GMM estimation techniques in which we always control for year and province dummies, treating some of the covariates as potentially endogenous (i.e. the lagged value of tax evasion, Total, Current and Capital spending per capita and the GDP per capita),^6^ since the system GMM is considered more efficient than the difference GMM.^7^

Moreover, the two-step procedure, in a system GMM setting, is more suitable as it leads to a consistent (as the one-step procedure) and asymptotically efficient estimator. However, as our sample size is small, although the number of cross-section units (*provinces*) is larger than the time series units (*years*), the one-step system GMM is preferred compared to the two-step technique. We also collapse the number of instruments, as suggested by Roodman (2009), to avoid redundancy in the instruments used. Furthermore, we use robust estimator of the covariance matrix of the parameter estimates with the resulting standard error estimates that are consistent in the presence of any pattern of heteroskedasticity and autocorrelation within panels, and we apply the backward orthogonal deviations that replace instruments with their deviations from past means in order to avoid any potential correlation between the instruments and the error term.

Results are displayed in Table 3, where we replicate the same specifications as those of Table 2. All in all, we can notice that a meaningful correction for the dynamic panel and endogeneity bias does not alter the qualitative nature of our main results. In fact, comparing the estimates to those reported in Table 2, it is clear they attract the same sign, although they are bigger in terms of magnitude, highlighting in turn how OLS estimates are downward bias. In particular, we observe a negative and significant effect of government size, as measured by both total and capital expenditures per capita, on tax evasion. Also, the inclusion of covariates does not affect our findings.

**Table 3:** Tax evasion and spending

Variables	Tax evasion (1)	Tax evasion (2)	Tax evasion (3)	Tax evasion (4)	Tax evasion (5)	Tax evasion (6)
Tax evasion $(t-1)$	0.930*** (0.127)	0.590*** (0.139)	0.934** (0.112))	0.798*** (0.0734)	0.361 (0.297))	0.540*** (0.107)
Total expenditures	− 0.0658* (0.0348)	− 0.1310* (0.0763)				
Current expenditures			− 0.0262 (0.0211)	0.0139 (0.0189)		
Capital expenditures					− 0.0312* (0.0197)	− 0.0387*** (0.0122)
GDP per capita		− 0.0002 (0.0003)		− 0.0003** (0.0001)		− 0.0003** (0.0002)
Population/1000		− 0.0010* (0.0010)		− 0.0010 (0.0010)		− 0.0010* (0.0010)
%Elderly people		− 0.0170 (0.6550)		− 0.0361 (0.1340)		− 0.0175 (0.1440)
Employment rate		− 0.0068** (0.0034)		− 0.0001 (0.0007)		− 0.0019* (0.0011)
Province dummies	Yes	Yes	Yes	Yes	Yes	Yes
Year dummies	Yes	Yes	Yes	Yes	Yes	Yes
Arellano bond AR (2)	0.436	0.436	0.338	0.228	0.144	0.258
Hansen test	0.542	0.662	0.142	0.567	0.14	0.627
No. instruments	20	27	20	27	20	27
Observations	1483	1452	1483	1452	1483	1452
Number of provinces	106	106	106	106	106	106

GMM results

One-step system GMM estimates. We focus on the period 2001–2015. In each specification we control for year and province dummies. Standard errors are robust to heteroskedasticity and are clustered at the province level (shown in brackets). Significance at the 10% level is represented by *, at the 5% level by **, and at the 1% level by ***

Moreover, for all regressions at the bottom of Table 3, we report the results (*p-values*) of two key specification tests that are the * Hansen J*-test for instrument exogeneity and the * Arellano–Bond* test for second order autocorrelation. A significant * Hansen J*-statistic would indicate that some of the instruments are likely to be not exogenous. Similarly, a significant statistic for the * Arellano–Bond* autocorrelation test would indicate that some of our instruments are potentially correlated with the error term. However, as highlighted by the reported *p-values*, in both cases the statistics are never significant at any conventional levels.

In Table 4 we directly test Barone and Mocetti (2011) prediction about the relationship between tax evasion and provincial government efficiency. In fact, taxpayers’ discontent might rise as governments spend more than the level desired by citizens, or more than the resources collected through tax levy. In both cases local authorities work under inefficiency conditions, and following Gagliarducci and Nannicini (2013) we build two efficiency indicators for the management of the provincial government that take into account also the amount of revenues collected: the difference between total revenues and total spending (in natural log), i.e. the surplus (see column 1 and 2), and the speed of payment, measured by the ratio between paid and committed outlays (column 3 and 4) that is on average 1.12 and it has a standard deviation of 0.249.

GMM findings, in line with those found by Barone and Mocetti (2011) on tax morale at the municipal level in Italy, highlight that both measures of provincial government efficiency negatively impact tax evasion: the more the public administration is efficient the less is the propensity to evade taxes. Also the inclusion of controls in even specifications does not alter the results.

**Table 4:** Tax evasion and efficiency

Variables	Tax evasion (1)	Tax evasion (2)	Tax evasion (3)	Tax evasion (4)
Tax evasion $(t-1)$	0.975*** (0.0065)	0.348* (0.208)	0.978*** (0.0057)	0.556*** (0.105)
Surplus	− 0.0036*** (0.0010)	− 0.0709** (0.0331)		
Speed Payment			− 0.0112* (0.018)	− 0.0568** (0.0266)
Province dummies	Yes	Yes	Yes	Yes
Year dummies	Yes	Yes	Yes	Yes
Controls	No	Yes	No	Yes
Arellano Bond AR (2)	0.323	0.169	0.303	0.121
Hansen test	0.240	0.239	0.119	0.258
Observations	1459	1452	1459	1452
Number of provinces	106	106	106	106

GMM results

One-step system GMM estimates. We focus on the period 2001–2015. In each specification we control for year and province dummies. Standard errors are robust to heteroskedasticity and are clustered at the provincial level (shown in brackets). Significance at the 10% level is represented by *, at the 5% level by **, and at the 1% level by ***

### Capital and current spending: a closer look

In the previous subsection we have studied whether taxpayers’ behaviour is affected by the short-run (current spending) and/or the long-run (capital spending) investments implemented by provincial governments.

We have found that the downward variation in tax evasion is primarily driven by an increase in capital spending that, on the one hand, should be the type of spending citizens have a higher chance (and more time) to observe, and on the other hand, is less under the direct control of local policymakers/bureaucrats. However, the propensity to pay taxes might change according to the type of public goods provided by provincial governments. At this aim, we further exploit information about spending coming from local balance sheets and measure our main variable of interest as expenditures in (1) police/security, (2) justice, (3) economic development, (4) welfare/health, (5) education, and (6) transport, respectively. All the above variables are normalized by the population size and built in logarithm.

**Table 5:** Tax evasion and current spending

Variables	Tax evasion (1)	Tax evasion (2)	Tax evasion (3)	Tax evasion (4)	Tax evasion (5)	Tax evasion (6)
Tax evasion ($t-1$)	0.779*** (0.0686)	0.745*** (0.0747)	0.788*** (0.0677)	0.770*** (0.0706)	0.764*** (0.0749)	0.759*** (0.0664)
Police/security expenditures	− 0.0238 (0.0267)					
Justice expenditures		− 0.0370 (0.0525)				
Development expenditures			− 0.0273 (0.0237)			
Welfare/health expenditures				− 0.0024 (0.0155)		
Education expenditures					− 0.0071 (0.0126)	
Transport expenditures						− 0.0867** (0.0409)
Province/year dummies	Yes	Yes	Yes	Yes	Yes	Yes
Arellano bond AR(2)	0.186	0.151	0.167	0.183	0.166	0.138
Hansen test	0.364	0.353	0.327	0.367	0.448	0.369
Controls	Yes	Yes	Yes	Yes	Yes	Yes
Observations	1452	1452	1452	1452	1452	1452
Number of provinces	106	106	106	106	106	106

GMM results

One-step system GMM estimates. We focus on the period 2001–2015. In each specification we control for year and province dummies. Standard errors are robust to heteroskedasticity and are clustered at the provincial level (shown in brackets). Significance at the 10% level is represented by *, at the 5% level by **, and at the 1% level by ***

All in all, our results are similar to those previously presented. In particular, we find that increasing police/security, welfare/health and transport spending leads to a downward shift in tax evasion (see Table 6), whereas no statistically significant impact is detected for each component of current expenditures (see Table 5).

**Table 6:** Tax evasion and capital spending

Variables	Tax evasion (1)	Tax evasion (2)	Tax evasion (3)	Tax evasion (4)	Tax evasion (5)	Tax evasion (6)
Tax evasion $(t-1)$	0.713*** (0.0907)	0.782*** (0.135)	0.703*** (0.0842)	0.582*** (0.0936)	0.705*** (0.0751)	0.674*** (0.0933)
Police/security expenditures	− 0.201* (0.101)					
Justice expenditures		− 0.121 (2.279)				
Development expenditures			− 0.0412 (0.0506)			
Welfare/health expenditures				− 0.401** (0.169)		
Education expenditures					− 0.065 (0.862)	
Transport expenditures						− 0.116* (0.0743)
Province/year dummies	Yes	Yes	Yes	Yes	Yes	Yes
Arellano bond AR (2)	0.156	0.136	0.187	0.183	0.949	0.133
Hansen test	0.370	0.479	0.376	0.367	0.369	0.342
Controls	Yes	Yes	Yes	Yes	Yes	Yes
Observations	1452	1452	1452	1452	1452	1452
Number of provinces	106	106	106	106	106	106

GMM results

One-step system GMM estimates. We focus on the period 2001–2015. In each specification we control for year and province dummies. Standard errors are robust to heteroskedasticity and are clustered at the provincial level (shown in brackets). Significance at the 10% level is represented by *, at the 5% level by **, and at the 1% level by ***

What emerges from our results is that public spending has an impact on tax evasion, but it turns out to be negative and significant only for long-term expenditures, and in particular for those borne to provide services in health, security and transportation, whereas no significant effect arises when considering current spending. Some deeper considerations are therefore necessary.

Mario Draghi—during the 2020 Meeting (Special Edition) held in Rimini last August and referring to the current economic crisis caused by the Covid-19 pandemic - made a distinction between “good” and “bad” debt; hence, between “good” and “bad” spending. “Good” spending goes towards investments in research and innovation, environment and energy, as well as toward reforms in education and research, public administration and justice. “Bad” spending mainly consists of the distribution of subsidies, benefits, early pensions, alms and so on. Although identifying current expenditures as “bad” and capital expenditures as “good” sounds too rough, this distinction seems appropriate to explain our results and, more importantly, sheds light on how public expenditure is perceived by citizens.

In fact, it is well known that current spending has been often used by politicians to increase public consensus, whereas capital expenditure should be less perceived as it produces effects in the long-run and could/should mainly benefit future generations. On this point, Cerniglia et al. (2017) found that the composition of decentralized public expenditure matters for growth in the following way: the effect of capital expenditure on growth is positive, whereas decentralized current spending tends to have a negative effect. Similarly, we find that only capital spending for some sectors is able to make a break into taxpayers’ tendency to evade taxes. Why?

To answer this question it is worthwhile to remind ourselves who gains from “bad” expenditure and who can evade. Certainly bad expenditure benefits public workers as it increases bureaucracy, unemployed people who receive subsidies, and workers enjoying an earlier retirement. In other words, current spending cannot make a substantial contribution in explaining the change in tax evasion if all these categories of citizens/taxpayers are also the main recipients of current expenditure in most sectors, including health, security, education, and transportation: indeed, according to the Italian Public Accounts (IPA) Observatory (Catholic University of Milan) the least evaded tax in 2017 is personal income tax on employees (gap of 2.9%). This descriptive evidence explains why current expenditure in any of the analyzed sectors does not significantly affect taxpayers’ behaviour.

On the other hand, more than half of the amount of taxes evaded (69.1 billion out of 108.1) in the same year relates to VAT and personal income tax on self-employed workers and companies. Furthermore, the latter tax is also the one that contributes the most to the gap: approximately 2/3 of the amount due would be evaded (whereas the gap for the other most evaded taxes is between 20 and 27%).

If companies and self-employed workers and companies cannot benefit from an increase of current expenditure in any sector as it mainly produces its effects on retired or unemployed people, they do benefit from public investments in key sectors of general interest, like infrastructures, health and security: this might explain the negative and significant relationship between capital expenditure for these sectors and tax evasion. Conversely, capital expenditure in other sectors referring to specific interests and groups, like justice and education, produces a little impact on self-employed workers and companies and turns out not to produce any effect on tax evasion.

## Heterogeneity

The channels through which public spending affects taxpayers’ behaviour may work dissimilarly in different parts of Italy. We are indeed considering a country that is very heterogeneous in terms of economic and social conditions, with the northern part being richer and endowed with higher social capital compared to the south. In addition, the north is characterized by a low level of tax evasion, as shown in Fig. 1, and provincial governments spend on average more compared to those located in the south. To investigate whether the relationship between government size and tax evasion is heterogeneous in the two parts of the country we have split the sample according to a dummy variable taking the value of 1 if the province belongs to the south and 0 otherwise.

**Table 7:** Tax evasion and public spending: south vs north

Variables	Tax evasion (1) north	Tax evasion (2) south	Tax evasion (3) north	Tax evasion (4) south	Tax evasion (5) north	Tax evasion (6) south
Tax evasion ($t-1$)	0.900*** (0.0867)	0.895*** (0.0818)	0.987*** (0.0822)	0.891*** (0.107)	0.654*** (0.145)	0.922*** (0.0808)
Total expenditures	− 0.0374** (0.0159)	0.0049 (0.0240)				
Current expenditures			− 0.0154 (0.0131)	0.0234 (0.0454)		
Capital expenditures					− 0.0311** (0.0140)	0.0033 (0.0204)
Province dummies	Yes	Yes	Yes	Yes	Yes	Yes
Year dummies	Yes	Yes	Yes	Yes	Yes	Yes
Arellano bond AR (2)	0.223	0.538	0.237	0.578	0.255	0.530
Hansen test	0.251	0.277	0.216	0.279	0.279	0.146
Controls	Yes	Yes	Yes	Yes	Yes	Yes
Observations	943	509	943	509	943	509
Number of provinces	69	37	69	37	69	37

GMM results

One-step system GMM estimates. We focus on the period 2001–2015. In each specification we control for year and province dummies and for the full set of controls. Standard errors are robust to heteroskedasticity and are clustered at the provincial level (shown in brackets). Significance at the 10% level is represented by *, at the 5% level by **, and at the 1% level by ***

Results are displayed in Table 7. We measure government size as total (column 1–2), current (column 3–4) and capital expenditures per capita (column 5–6) respectively. We show that the impact of government size on tax evasion is in fact heterogeneous: the coefficient attached to total public spending is negative and statistically significant only for provinces located in the north. In particular, an increase of public spending per capita by 1% reduces tax evasion by 3.74%, whereas in the south the variable of interest attracts a positive but not statistically significant coefficient. Moreover, the difference between the coefficient attached to total spending is significant among groups (north vs south) at the 5% level. The same results are true when the focus is on capital spending per capita, while we do not find any heterogeneous impact of current expenditures on tax evasion in the two main areas under scrutiny.

We are aware that the heterogeneous effect is very hard to be interpreted as causal, as many other factors might differ between south and center-north, and in turn, we can never fully attribute the differences found to a specific dimension. Nonetheless, our findings are only suggestive and relate to Gordon’s (1989) intuition: in poor areas, as the south of Italy, where the level of spending is usually low compared to that registered in richer areas, individuals do not reduce the propensity to evade taxes as they feel the government intervention, also through an increase in public spending, is not enough to revive the fortunes of their economy, hoping in a better provision of public goods.

To deeply investigate this heterogenous effect between provinces located in the south and in the north of the country, we split the sample according to the median of the “historical” level of public goods provision”, measured by the total amount of money invested by provincial governments in public works in 2001 (data provided by ISTAT). Results are displayed in Table 8 in which for space reasons we focus on capital spending per capita only, although the same findings hold true also for total spending.

Overall, we highlight a negative and significant impact of capital spending on taxpayers’ behaviour only for those provinces characterized by a high “historical” level of public goods provision. Furthermore, in columns (3)–(4) and (5)–(6) we split the sample based on the number of works involved in the provision of two particular public goods (median), i.e. the construction/maintenance of streets and hospitals, respectively. Again, we find that increasing capital spending negatively affects tax evasion only in those areas where the intervention of provincial governments is strong, whereas for those provinces below the median the impact of interest is not significant at any conventional levels.

**Table 8:** Tax evasion, public spending and intervention of governments

Variables	Tax evasion (1) > 50th public works	Tax evasion (2) < 50th public works	Tax evasion (3) > 50th streets	Tax evasion (4) < 50th streets	Tax evasion (5) > 50th hospitals	Tax evasion (6) < 50th hospitals
Tax evasion $(t-1)$	0.454*** (0.108)	0.662*** (0.151)	0.527** (0.227)	0.544*** (0.108)	0.514*** (0.127)	0.625*** (0.178)
Capital expenditures	− 0.0399** (0.0166)	− 0.0102 (0.0153)	− 0.0473* (0.0281)	− 0.0171 (0.0113)	− 0.0404*** (0.0142)	− 0.0379 (0.0243)
Province dummies	Yes	Yes	Yes	Yes	Yes	Yes
Year dummies	Yes	Yes	Yes	Yes	Yes	Yes
Arellano bond AR (2)	0.535	0.214	0.149	0.917	0.254	0.617
Hansen test	0.225	0.195	0.897	0.149	0.136	0.159
Controls	Yes	Yes	Yes	Yes	Yes	Yes
Observations	732	720	759	693	731	721
Number of provinces	54	52	56	50	54	52

GMM results

One-step system GMM estimates. We focus on the period 2001–2015. In each specification we control for year and province dummies and for the full set of controls. Standard errors are robust to heteroskedasticity and are clustered at the provincial level (shown in brackets). Significance at the 10% level is represented by *, at the 5% level by **, and at the 1% level by ***

Moreover, the effect of public spending on tax evasion might depend on the level of satisfaction citizens have in the area they live. We exploit information at regional level provided by ISTAT on the percentage of households complaining about some goods and services provided by regional governments, i.e. traffic and condition of streets: these variables can be seen as direct proxies of the quality of public goods. In particular, we build two dummy variables that are equal to 1 if the percentage of families complaining is above the median and zero otherwise, and split the sample accordingly. As highlighted in Table 9, capital spending (the same results hold true for total spending) has a negative impact on tax evasion only in areas characterized by a low level of complaints, suggesting that citizens’ perception about the quality of public goods provided is an important driver mitigating the impact of public spending on tax evasion.

**Table 9:** Tax evasion, public spending and quality of public goods

Variables	Tax evasion (1) >50th traffic	Tax evasion (2) < 50th traffic	Tax evasion (3) > 50th streets	Tax evasion (4) < 50th streets
Tax evasion $(t-1)$	0.700*** (0.132)	0.671*** (0.151)	0.791*** (0.106)	0.663*** (0.0969)
Capital expenditures	− 0.0098 (0.0173)	− 0.0202* (0.0109)	− 0.0059 (0.0073)	− 0.0322* (0.0203)
Province dummies	Yes	Yes	Yes	Yes
Year dummies	Yes	Yes	Yes	Yes
Arellano bond AR (2)	0.615	0.148	0.551	0.378
Hansen test	0.217	0.128	0.165	0.187
Controls	Yes	Yes	Yes	Yes
Observations	719	733	790	662
Number of provinces	52	54	57	49

GMM results

One-step system GMM estimates. We focus on the period 2001–2015. In each specification we control for year and province dummies and for the full set of controls. Standard errors are robust to heteroskedasticity and are clustered at the provincial level (shown in brackets). Significance at the 10% level is represented by *, at the 5% level by **, and at the 1% level by ***

Finally, we check whether the negative impact of public spending on tax evasion is heterogeneous according to the median value of a corruption index—built at regional level over the period 2001–2013—as measured by the number of convicted people for different crimes, such as embezzlement, over the total number of cases registered in Italy. Unfortunately, we are not able to gather data on corruption at provincial level, and this is the reason why we cannot add this variable among our full set of controls. Furthermore, not only data on corruption at province level, but also information on political trust at any level are unavailable from ISTAT.

**Table 10:** Tax evasion, public spending and corruption

Variables	Tax evasion (1) < median	Tax evasion (2) > median	Tax evasion (3) < median	Tax evasion (4) > median	Tax evasion (5) < median	Tax evasion (6) > median
Tax evasion $(t-1)$	0.694*** (0.0483)	0.530*** (0.0876)	0.632*** (0.0806)	0.570*** (0.186)	0.722*** (0.0517)	0.544*** (0.0880)
Total expenditures	− 0.0133** (0.0066)	− 0.0036 (0.0106)				
Current expenditures			− 0.0119 (0.0197)	− 0.0037 (0.0107)		
Capital expenditures					− 0.0128* (0.0068)	− 0.0029 (0.0108)
Province and year dummies	Yes	Yes	Yes	Yes	Yes	Yes
Arellano bond AR (2)	0.148	0.188	0.109	0.105	0.243	0.199
Hansen test	0.181	0.425	0.124	0.208	0.192	0.864
Controls	Yes	Yes	Yes	Yes	Yes	Yes
Observations	614	627	614	627	614	627

GMM results

One-step system GMM estimates. We focus on the period 2001–2013. In each specification we control for year and province dummies and in even columns for the full set of controls. Errors are robust to heteroskedasticity and are clustered at the provincial level (shown in brackets). Significance at the 10% level is represented by *, at the 5% level by **, and at the 1% level by ***

Results are reported in Table 10. In each specifications we add the full set of controls along with province and year fixed effects. Overall, they are in line with our expectations: an increase in the public spending leads to a downward shift in tax evasion only in those provinces characterized by a low level of corruption.

## Robustness checks

As a first robustness of our empirical exercise, we check whether our results change when we implement a different estimation procedure. In Table 11 we present findings coming from the implementation of a difference-GMM (columns 1, 2 and 3) and a two-step system GMM (columns 4, 5 and 6), measuring our variable of interest as total, current and capital expenditures per capita (in natural log). Overall, results are very similar in terms of sign and magnitude to those previously presented. Also, the p-values of two key specification tests for instrument exogeneity and second order autocorrelation, displayed at the bottom of the table, are always larger than 0.10.

**Table 11:** Tax evasion and public spending: Diff-GMM and Two-step system

Variables	Tax evasion (1) Diff-GMM	Tax evasion (2) Diff-GMM	Tax evasion (3) Diff-GMM	Tax evasion (4) Two-step sys	Tax evasion (5) Two-step sys	Tax evasion (6) Two-step sys
Tax evasion $(t-1)$	0.482*** (0.0823)	0.652*** (0.0650)	0.470*** (0.0998)	0.579*** (0.155)	0.842*** (0.107)	0.566*** (0.138)
Total expenditures	− 0.0847*** (0.0211)			− 0.138** (0.0644)		
Current expenditures		− 0.0268 (0.0277)			0.0098 (0.0219)	
Capital expenditures			− 0.0157** (0.0068)			− 0.0354** (0.0153)
Province dummies	Yes	Yes	Yes	Yes	Yes	Yes
Year dummies	Yes	Yes	Yes	Yes	Yes	Yes
Arellano bond AR(2)	0.191	0.128	0.181	0.191	0.228	0.236
Hansen test	0.215	0.251	0.214	0.662	0.279	0.167
Controls	Yes	Yes	Yes	Yes	Yes	Yes
Observations	1346	1346	1346	1452	1452	1452
Number of provinces	106	106	106	106	106	106

GMM results

Diff-GMM (column 1, 2 and 3) and Two-step system GMM (column 4, 5 and 6) estimates. We focus on the period 2001–2015. In each specification we control for year and province dummies and for the full set of controls. Standard errors are robust to heteroskedasticity and are clustered at the provincial level (shown in brackets). Significance at the 10% level is represented by *, at the 5% level by **, and at the 1% level by ***

Moreover, as highlighted in Fig. 1 tax evasion sharply increases after the worldwide financial crisis started in 2007. In the main specifications we only controlled for year dummies to take into account common economic shocks that have affected all the provinces. As a further robustness, we also want to consider that the financial crisis has potentially affected taxpayers’ behaviour in a different way among provincial areas. In Table 12 we add among controls the interaction between province FE and a dummy variable taking the value 1 for years after 2007 and 0 otherwise. In columns (1)–(3) we present FE estimates, whereas in the remaing specifications we display findings coming from the implementation of a one-step system GMM. All in all, controlling for the financial crisis effect does not alter our findings.

**Table 12:** Tax evasion, public spending and the financial crisis in 2007

Variables	Tax evasion (1) FE	Tax evasion (2) FE	Tax evasion (3) FE	Tax evasion (4) one-step sys	Tax evasion (5) one-step sys	Tax evasion (6) one-step sys
Tax evasion $(t-1)$	0.437*** (0.0287)	0.445*** (0.0292)	0.445*** (0.0292)	0.377*** (0.0859)	0.477*** (0.0682)	0.422*** (0.0746)
Total expenditures	− 0.0198** (0.0082)			− 0.106* (0.0620)		
Current expenditures		− 0.0143 (0.0104)			− 0.0084 (0.0348)	
Capital expenditures			− 0.0096** (0.0042)			− 0.0223** (0.0100)
Province dummies	Yes	Yes	Yes	Yes	Yes	Yes
Year dummies	Yes	Yes	Yes	Yes	Yes	Yes
Controls	Yes	Yes	Yes	Yes	Yes	Yes
Observations	1452	1452	1452	1452	1452	1452
Number of provinces	106	106	106	106	106	106

One-step system GMM results

Province FE (column 1, 2 and 3) and One-step system GMM (column 4, 5 and 6) estimates. We focus on the period 2001–2015. In each specification we control for year and province dummies and for the full set of controls. Standard errors are robust to heteroskedasticity and are clustered at the provincial level (shown in brackets). Significance at the 10% level is represented by *, at the 5% level by **, and at the 1% level by ***

Furthermore, it is well known that the north subsidizes the south. Thus, provinces in the south are expected to consume public goods and services partly financed by the north. This must affect citizens’ evaluation of public goods provision and that of people living in the northern part of Italy. To solve this issue we also add among controls the total amount of current and capital transfers (per capita) received by regions or by the central government.

**Table 13:** Tax evasion, public spending and transfers

Variables	Tax evasion (1)	Tax evasion (2)	Tax evasion (3)	Tax evasion (4) north	Tax evasion (5) south
Tax evasion $(t-1)$	0.641*** (0.0892)	0.600*** (0.0928)	0.794*** (0.0909)	0.831*** (0.0891)	0.884*** (0.108)
Total expenditures	− 0.0369*** (0.0138)			− 0.0335*** (0.0119)	− 0.0166 (0.0231)
Capital expenditures		− 0.0252** (0.0098)			
Current expenditures			0.0045 (0.0206)		
Total capital transfers	− 0.0117 (0.0107)			− 0.0269** (0.0118)	− 0.0020 (0.0151)
Total current transfers	0.0207** (0.0092)			0.0225** (0.0106)	0.0044 (0.0186)
Regional capital transfers		− 0.0259** (0.0127)			
State capital transfers		0.0079 (0.0124)			
Regional current transfers			0.0044 (0.0077)		
State current transfers			0.0047 (0.0068)		
Province/year dummies	Yes	Yes	Yes	Yes	Yes
Controls	Yes	Yes	Yes	Yes	Yes
Observations	1452	1452	1452	1452	1452
Number of provinces	106	106	106	106	106

One-step system GMM results

One-step system GMM estimates. We focus on the period 2001–2015. In each specification we control for year and province dummies and for the full set of controls. All the transfers measures are considered as endogenous

In particular, in column (1) of Table 13 where the government size is measured by the total spending per capita, we control for the total capital and current transfers per capita. In column (2) and (3), in which the main variable of interest is the current and capital spending, we split transfers into those granted by regions and central government, respectively. Furthermore, in the last two specifications, we replicate estimation presented in Table 7. Overall, findings do not change, reassuring us that the heterogenous impact of government size on tax evasion presented in Table 7 do not simply come from the fact that citizens’ evaluation of public goods provision is affected by the amount of transfers received.

As a further robustness of our empirical exercise, we introduce a test proposed by Lopez and Weber (2017) which implements a procedure recently developed by Dumitrescu and Hurlin (2012) (hereafter DH) in order to test for Granger causality in panel datasets.^8^ In particular, we verify whether government size, measured by total and capital spending per capita (in log) respectively, Granger-causes tax evasion or vice versa. In fact, as explained before, the GMM estimates (correlations) previously presented, although robust, might be affected by a reverse causality issue. We report the results of the test in Table 14 in which we present the Wald statistic and p-values (in brackets). In column (1) and (2) total and capital spending per capita Granger-cause tax evasion, whereas in column (3) and (4) we never reject the null hypothesis (p-values always above 0.10) by which tax evasion Granger-causes the two measures of government size. All in all, our findings highlight a *unidirectional* Granger-causality from government size to tax evasion.

**Table 14:** Tax evasion and public spending

Variables	Tax evasion (1)	Tax evasion (2)	Total expenditures per capita (ln) (3)	Capital expenditures per capita (ln) (4)
Total expenditures per capita (ln)	3.970 (0.005)			
Capital expenditures per capita (ln)		5.5350 (0.000)		
Tax evasion			1.3337 (0.6906)	1.3736 (0.5565)
Lags	min(AIC)	min(AIC)	min(AIC)	min(AIC)

Granger causality test

Granger causality test proposed by Lopez and Weber (2017). In each column we report the Wald statistic and p-values in round brackets. The number of lags of the series are chosen such that the average Akaike information criterion for the set of regressions is minimized

As a final robustness check we implement an IV strategy in which we use the level of spending per capita in log—i.e. total, current and capital expenditures per capita depending on the specifications) borne by province governments in 1998 as an instrument for government size. It is necessary to stress that data about governments’ budgets are available since 1998 and that the use of our instrument comes with the cost of reducing the sample period. In particular, by focusing on the period 2006–2015—rather than 2001–2015—we stay far away from the historical level of spending borne in 1998, and we are sure that our instrument is likely to be valid since it is highly correlated with the level of spending borne by province governments from 2006 onwards, and taxpayers’ behaviour should not be directly affected—starting from 2006—by the level of expenditure borne in 1998. Results are reported in Table 15 and are very similar to those highlighted when the one-step system GMM is adopted. In addition, from Panel (b) in which the First stage findings are shown, the F- statistic is well above 10, suggesting that our estimates do not suffer from the issue of weak instrument.

**Table 15:** Tax evasion and public spending

Variables	Tax evasion (1)	Tax evasion (2)	Tax evasion (3)	Tax evasion (4)	Tax evasion (5)	Tax evasion (6)
*Panel (a): 2SLS*						
Tax evasion $(t-1)$	0.604*** (0.0594)	0.549*** (0.0302)	0.624** (0.123))	0.431*** (0.0618)	0.528*** (0.0840))	0.517*** (0.0364)
Total expenditures	− 0.0341*** (0.0118)	− 0.0179** (0.0086)				
Current expenditures			− 0.0556 (0.0452)	− 0.0611 (0.0429)		
Capital expenditures					− 0.0648*** (0.0249)	− 0.0261** (0.0118)
Constant	0.308*** (0.0792)	0.368*** (0.0292)	0.383** (0.155)	0.699*** (0.101)	0.311*** (0.0863)	0.374*** (0.0326)
*Panel (b): First stage*						
Instrument	0.586*** (0.049)	0.679*** (0.039)	0.701*** (0.061)	0.861*** (0.048)	0.509*** (0.066)	0.756*** (0.077)
*p*-value	0.000	0.000	0.000	0.000	0.000	0.000
F-stat	139.62	297.01	134.87	322.10	60.10	96.13
Controls	No	Yes	No	Yes	No	Yes
Province dummies	Yes	Yes	Yes	Yes	Yes	Yes
Year dummies	Yes	Yes	Yes	Yes	Yes	Yes
Observations	999	996	999	996	999	996
R-squared	0.963	0.965	0.969	0.976	0.950	0.964

IV approach

2SLS estimates. In columns (1) and (2) the outcome variable is measured by Total expenditures per capita, in columns (3) and (4) by Current Expenditures per capita and in columns (5) and (6) by Capital Expenditures per capita. We focus on the period 2006-2015. In each specification we control for year and province dummies. In even columns we also control for the full set of controls. Standard errors are robust to heteroskedasticity and are clustered at the provincial level (shown in brackets). Significance at the 10% level is represented by *, at the 5% level by **, and at the 1% level by ***

## Concluding remarks

Tax evasion is increasingly considered as a social disease in every country, and reducing its negative impact on the national economy as a whole is a priority for policy-makers. Unfortunately, the government may involuntarily contribute to encourage citizens in evading from the payment of taxes, especially when they feel unsatisfied about how government spends their money.

A large literature has focused on the relationship between tax evasion and public expenditures, as a proxy of government size, emphasizing its potential effect on tax payers’ motivations (Alm et al. 1992; Webley et al. 1991; Barone and Mocetti 2011). Our paper falls in this field and focuses on Italy, an European country characterized, on the one hand, by geographical heterogeneity in both economic conditions and social welfare and, on the other hand, by a very high level of tax evasion.

Using provincial data on tax evasion provided by the Italian Revenue Agency and information on local public expenditures provided by the Ministry of Internal Affairs over the period 2001–2015 we have tested whether government size, as measured by public spending per capita, affects tax evasion in Italy.

First, we have implemented a dynamic OLS estimation with fixed effects, to take into account time-invariant characteristics of provinces affecting both public spending and taxpayers’ behaviour, showing that the level of total and capital spending per capita is negatively correlated to tax evasion. To explain this result we have conjectured that taxpayers perceive the increase in public expenditure as a symptom that the government is efficiently spending their money, and consequently they feel satisfied on average, especially when the upward change regards those expenses borne for a long-term management of the public “ purse” that are less under control of bureaucrats.

Second, we have applied a system-GMM technique aimed at solving potential endogeneity issues affecting our model, and found qualitatively similar results. All in all, our data confirm our hypothesis, as using disaggregated data on each component of capital expenditures we find that in wide sectors of expenditures, such as security, welfare and transport, tax evasion decreases due to an upward change in the level of spending. Finally, our findings show that the public spending effect is also heterogeneous among provinces located in the north and in the southern part of Italy, suggesting that in poorer areas, usually characterized by a low level of spending, citizens’ willingness to pay taxes is not affected by an increase in public spending, since it is perceived the government is not involved enough to enhance the economic conditions of the area.

Nonetheless, evaluating the public spending effect using more disaggregated data in terms of tax evasion, distinguishing between personal income tax, corporate income tax and value-added tax for instance, would be helpful for policy-makers as it could provide more accurate predictions on how government size affects tax evasion, and is left for future research.

## Appendix A: Theoretical model

### Model

The game is played for two periods ($t=1,2$) by the local Government (*G*) and taxpayers (*T*). In period 1 *G* sets the budget $\left\{ \tau ,e,q\right\}$, where $\tau$ represents the amount of taxes coming from the tax levy (revenues), *e* is the level of public expenditure borne to provide valuable public goods, and *q* is the quality of *e*. Note that *e* measures the total amount of public expenditure. Without loss of generality, we assume that $e=\left\{ {\bar{e}},e_{H}\right\}$ with ${\bar{e}}<e_{H}$. We also assume that ${\bar{e}}$ corresponds to the amount of public expenditure necessary to provide an essential level of public goods without any further consideration about quality. Moreover, we say that $q({\bar{e}})=\phi$ to indicate that quality is empty. If $e=e_{H}$ then *q* does matter, because there is an extra-expenditure that *G* can use either to enhance the quality of public goods or to finance patronage and bureaucracy. Accordingly, we say $q(e_{H})=\left\{ b,t,s\right\}$, where *b* means bureaucracy, *t* stands for spending of general interest (transport, infrastracture, health, police for instance) and *s* stands for spending of specific interest (schooling, justice, etc.). Furthemore, we refer to $q(e_{H})=\left\{ t,s\right\}$ as high quality spending, and to $q(e_{H})=b$ as bad quality spending, respectively.

In period 2 *G* can decide to increase/decrease only *e* and the level of revenues $\tau ,$ but cannot modify the quality *q* of the extra-expenditure borne in the previous period ($e=e_{H}$) if $q\ne \phi$.

We define *c*(*e*, *q*) as the cost for *G* to provide *e*, assuming that $c(e_{H},b)<c({\bar{e}},\phi )<c(e_{H},s)<c(e_{H},t)$. To simplify notation, we say $c(e_{H},b)=c_{B}$, $c({\bar{e}},\phi )={\bar{c}}$, $c(e_{H},s)=c_{S}$, and $c(e_{H},t)=c_{T}$. Note that the cost function reflects the return of *e* for *G*, mainly in terms of electoral support: given $\tau$, *G* prefers to provide $e_{H},b$ over $e_{H},s$ over ${\bar{e}},\phi$ over $e_{H},t$. Each period *G*’s utility is therefore given by $U^{G}=\tau -c(e,q).$

As far as (*T*)axpayers are concerned, if $q\ne s$ they hold homogeneous preferences over (*e*, *q*) and evaluate a given policy as follows: $U^{T}({\bar{e}},\phi )={\bar{u}}$, $U^{T}(e_{H},b)=u_{L}$, and $U^{T}(e_{H},t)=u_{H}$ with $u_{L}<{\bar{u}}<u_{H}$ If $q=s$, then we have $T=\alpha T_{S}+(1-\alpha )T_{T}.$ The former category values *s* as important as *t*, whereas the latter category is not interested in *s* and values it as no extra-expenditure is provided. Accordingly, we say that $U^{T_{S}}(e_{H},s)=u_{H}$ and $U^{T_{T}}(e_{H},s)={\bar{u}}$. In period 1 *T* also differ in terms of a priori beliefs on *G*’s policy: we say that $\gamma T$ are optimistic and believe that *G* provides their first choice quality, whereas ($1-\gamma )T$ are skeptical with *G* because they believe that $q=b$ . In period 2 *T* observe the quality they prefer the most, i.e. all $T_{T}$ will observe $q=t$ and all $T_{S}$ will observe $q=\left\{ t,s\right\}$ whereas just a proportion $\lambda <1$ observes other qualities, with $(1-\lambda )$ *T* keeping original beliefs. Conversely, if in period 1 *G* has provided ${\bar{e}}$, *T* cannot experience any *q* because by assumption $q({\bar{e}})=\phi$. Finally, we assume that $T_{S}$ are provided with low income (below the median of the income distribution, for instance), $Y_{L}$; whereas $T_{T}$ are provided with high income (above the median), $Y_{H}$.

Note that *T*’s evaluation corresponds to the maximum level of taxes they are willing to pay, so that ${\bar{u}}\equiv {\bar{\tau }}Y_{i}$, $u_{L}\equiv \tau _{L}Y_{i}$ and $u_{H}\equiv \tau _{H}Y_{i}$, where $i=\left\{ L,H\right\}$ and $0<\varvec{\tau }_{L}<{\bar{\mathbf {\tau }}}<$ $\varvec{\tau }_{H}<1$ is the tax rate. *T*’s utility function is therefore given by $u(e,q)-\tau Y$. *T* decide to pay taxes iff $U^{T}\ge 0$ and evade otherwise. To simplify notation we will use $\tau$ instead of *u* and define $\varvec{\tau } _{L}\equiv \alpha \tau _{L}Y_{L}+(1-\alpha )\tau _{L}Y_{H}$; $\varvec{\tau } _{H}\equiv \alpha \tau _{H}Y_{L}+(1-\alpha )\tau _{H}Y_{H}$; and $\mathbf { {\bar{\tau }}}\equiv \alpha {\bar{\tau }}Y_{L}+(1-\alpha ){\bar{\tau }}Y_{H}$.

**Efficiency condition:** $\varvec{\tau }_{L}-c_{B}<\mathbf { {\bar{\tau }}}-c_{S}<{\bar{\mathbf {\tau }}}-{\bar{c}}<\varvec{\tau }_{H}-c_{T}$ meaning that providing $(e_{H},g)$ is the most efficient policy, the second best being $({\bar{e}},\phi )$ the third being $(e_{H},v)$, and the last best being $(e_{H},b)$.

### Results and predictions

#### Proposition 1

(*b**-equilibrium): If* $\gamma >\max \left[ 1-\frac{2(c_{T}-c_{B})}{ (1-\lambda )\varvec{\tau }_{H}},\frac{2({\bar{\mathbf {\tau }}}-{\bar{c}}+c_{B})}{ (2-\lambda )\varvec{\tau }_{H}},\frac{\alpha \varvec{\tau } _{H}-2(c_{S}-c_{B})}{(1-\lambda )\varvec{\tau }_{H}}\right]$, *there exists a unique equilibrium in which* *G* *sets* $\left\{ \tau _{H},e_{H},b\right\}$*in both periods: optimistic* *T* (*proportion* $\gamma$) *pay taxes in period* 1, *whereas only unaware* *T* (*proportion*$\ 1-\lambda$) *pay in period* 2.

($\phi$-*equilibrium): If* $\gamma <\min \left\{ \frac{2({\bar{\mathbf {\tau }}}- {\bar{c}}+c_{B})}{(2-\lambda )\varvec{\tau }_{H}},\frac{2({\bar{\mathbf {\tau }}}- {\bar{c}}+c_{T})}{\varvec{\tau }_{H}}-1,\frac{2({\bar{\mathbf {\tau }}}-{\bar{c}} +c_{S})-\alpha \varvec{\tau }_{H}}{\varvec{\tau }_{H}[1+(1-\alpha )(1-\lambda )]},\frac{2({\bar{\mathbf {\tau }}}-{\bar{c}}+c_{B})-\varvec{\tau }_{L} }{\varvec{\tau }_{H}}\right\}$, *there exists a unique equilibrium in which* *G* *sets* $\left\{ {\bar{\tau }},{\bar{e}},\phi \right\}$ *in both periods with all* *T* *paying taxes.*

(*s**-equilibrium):* $\gamma \in \left[ \min \left\{ \frac{1}{(1-\lambda )}- \frac{2(c_{T}-c_{S})}{(1-\alpha )(1-\lambda )\varvec{\tau }_{H}},\frac{2( {\bar{\mathbf {\tau }}}-{\bar{c}}+c_{S})-\alpha \varvec{\tau }_{H}}{\varvec{\tau } _{H}[1+(1-\alpha )(1-\lambda )]}\right\} ,\max \left\{ \frac{{\bar{\mathbf {\tau }}}-{\bar{c}}+2c_{S}-c_{B}-\alpha \varvec{\tau }_{H}}{\varvec{\tau } _{H}(1-\alpha )(1-\lambda )},\frac{\alpha \varvec{\tau }_{H}-2(c_{S}-c_{B})}{ (1-\lambda )\varvec{\tau }_{H}}\right\} \right]$, *there exists a unique equilibrium in which* *G* *sets* $\left\{ \tau _{H},e_{H},s\right\}$ *in both periods with optimistic* *T* *(proportion* $\gamma$*) paying taxes in period* 1 *and only* $T_{S}$ *paying taxes in period* 2.

(*t**-equilibrium): If* $\gamma \in \left[ \max \left\{ \frac{2({\bar{\mathbf {\tau }}}-{\bar{c}}+c_{T})}{\varvec{\tau }_{H}}-1,\right\} ,\min \left\{ \frac{1}{ (1-\lambda )}-\frac{2(c_{T}-c_{B})}{(1-\lambda )\varvec{\tau }_{H}},\frac{1}{ (1-\lambda )}-\frac{2(c_{T}-c_{S})}{(1-\alpha )(1-\lambda )\varvec{\tau }_{H} }\right\} \right]$ *and* ${\bar{\mathbf {\tau }}}-{\bar{c}}+c_{T}-c_{B}<\mathbf { \tau }_{{\mathbf {H}}}-c_{T}$*, there exists a unique equilibrium in which* *G* *sets* $\left\{ \tau _{H},e_{H},t\right\}$ *in both periods, only optimistic* *T* *(proportion* $\gamma$*) pay taxes in period* 1 *and all* *T* *pay taxes in period* 2.

($\phi b$*-equilibrium):* *If* $\gamma \in \left[ \frac{{\bar{\mathbf {\tau }}}- {\bar{c}}+c_{B}}{\varvec{\tau }_{H}},\min \left\{ \frac{{\bar{\mathbf {\tau }}}- {\bar{c}}}{(1-\lambda )\varvec{\tau }_{H}},\frac{{\bar{\mathbf {\tau }}}-{\bar{c}} +2c_{S}-c_{B}-\alpha \varvec{\tau }_{H}}{\varvec{\tau }_{H}(1-\alpha )(1-\lambda )}\right\} \right]$, *and* ${\bar{\tau }}-{\bar{c}}+c_{T}-c_{B}>\tau _{H}-c_{T}$*, there exists a unique equilibrium in which* *G* *sets* $\left\{ {\bar{\tau }},{\bar{e}},\phi \right\}$ *in period* 1 *and* $\left\{ \tau _{H},e_{H},b\right\}$ *in period* 2 *with all* *T* *paying taxes in period* 1 *and only optimistic* *T* *(proportion* $\gamma$*) paying taxes in period* 2.

Proof is in the Appendix B.

#### Prediction 1

* Tax evasion decreases if the provision of public goods increases over time, also in terms of quality.*

See $\phi b$-equilibrium: *T* do not hold any a priori beliefs on *q* in period 1 because $e={\bar{e}}$, so they pay taxes. However, only optimistic *T* (proportion $\gamma$) trust *G* if $e=e_{H}$ in period 2 and pays taxes.

This simple model has the merit to verify taxpayers’ reactions to different policies implemented in period 1. What turns out of interest is therefore their behavior in period 2.

We are mainly interested in the behavior of those taxpayers who take into account to evade taxes, but only as a reaction of disappointment and/or unsatisfaction from the Government’s implemented policy; for this reason we have not included honesty as a discriminating category. This allows us to reach a compromise: if it is true that we consider people who care of society and public goods, at the same time our taxpayers do not like to be cheated. Accordingly, we have assumed that some taxpayers trust (does not trust) the Government in period 1 and believe that public expenditure is of high quality (or bad quality) unless they experience an opposite result: we have called them optimistic (pessimistic). Taxpayers also differ in their preferences: all of them like some expenditure (i.e. transportation), but only some of them equally like some other expenditure (i.e. schooling), e.g. citizens with young children, or those who work in the education sector. If expenditure is high, in period 2 all taxpayers experience the quality they prefer the most, but only some some of them (proportion $\lambda \ge 0$) will experience less valued qualities. The assumption is based on the consideration that taxpayers interested in a given expenditure, let’s say education, need or use that service (e.g. because they have young children) and are able to evaluate the quality of its provision; if they are not interested (e.g. single people or families without children), they may know about its provision only from other citizens, friends, colleagues or relatives, directly involved. Similarly, we assume that just some taxpayers (proportion $\lambda$) experience bad quality, i.e. bureaucracy.

Moreover, the model does not take into account the effects of tax evasion, like fines for evaders. Finally, our results follow from the assumption that *q*, the quality of the extra-expenditure, cannot be modified over time: *G* has to invest in a given sector, for instance, either transportation or schooling or in bureaucracy, and cannot switch money from one sector to another easily. To give a concrete example, once *G* has decided to hire new people in public offices in period 1 it cannot dismiss them in period 2.

We have decided to limit the analysis to a two-period model. Adding other periods is certainly possible, but would not change the final message significantly. First, periods should be limited to the lenght of a legislature because elections may affect the policy promised by the current Government (who aims to be re-elected) and the policy promised by its political opponents. Second, even forgetting this and other practical implications, if we add more periods we may guess the proportion of aware citizens increases period by period, with $\lambda$ possibly approching 1: this should make conditions for the *b*-equilibrium stricter and could also affect the $\phi$-equilibrium (with the Government switching to $(\tau _{H},e_{H},b)$ later than in period 2, and possibly in the last period).

We now try to draw some further predictions that are empirically testable. In the *b*-equilibrium taxpayers may decide to evade taxes in period 2 if they feel disappointed about how the Government has spent their money in period 1. This effect increases with the proportion $\lambda$ of aware taxpayers, i.e. citizens who have observed the quality *q* of the extra-expenditure. However, if the Government expects only a low proportion of taxpayers to become aware, and potentially disappointed, it can be pushed to set the highest available level of public expenditure: the higher cost borne by taxpayers in terms of taxes, however, is not compensated by a higher level of public services, but is rather used to finance patronage. The final result suggests an increase in the proportion of disappointed taxpayers $\lambda$, leading in turn to a high level of tax evasion.

An opposite outcome is found in the *t*-equilibrium if the proportion of aware taxpayers in period 2 is relatively high and the proportion of $T_{S}$ is relatively small: it is not profitable for the Govermnent to finance patronage or type *s*-spending as the amount of revenues coming from the tax levy would be very low, so financing *t* turns out to be the most profitable option.

We can sum up the intuitions of the *b*-equilibrium and the *t*-equilibrium in the following:

#### Prediction 2

*Tax evasion increases if public expenditure provided from the beginning is high and of bad quality, i.e. bureaucracy.*

#### Prediction 3

* Tax evasion decreases if public expenditure provided from the beginning is high and of general interest, i.e. transportation.*

Taxpayers prefer a high expenditure level, conditional on the Government providing their preferred quality in equilibrium. When the proportion of aware taxpayers is large enough, revenues in period 2 will decrease if the Government sets bad quality due to a massive evasion; moreover, when optimistic taxpayers are low enough, the Government will experience a very high evasion in period 1 if it sets a high level of expenditure. This is the reason supporting the existence of the $\phi$-equilibrium: if expenditure remains low, *G* cannot affect taxpayers’ behavior because they feel neither happy nor disappointed by definition.

We have assumed that high expenditure is of good quality if it is spent either for *t* or for *s*, but only a fraction of taxpayers (defined as $T_{S}$) like both equally, whereas the others (defined as $T_{T}$) strictly prefer the former over the latter. If the proportion of optimistic taxpayers is high enough to support high expenditure in period 1 and $T_{S}$ are numerous enough, the Governement may find profitable to set high expenditure from the beginning and to choose to finance spending of specific interest *s* (whose cost is cheaper than the cost for *t*): this leads to the *s* -equilibrium. It turns out that in period 1 only pessimistic taxpayers (proportion $1-\gamma$) evade taxes, whereas in period 2 only $T_{T}$ (proportion $1-\alpha$) feel disappointed and decide to evade. Since we have assumed that $T_{T}$ have high income, then tax evasion increases if $\frac{\lambda \gamma (1-\alpha )}{\alpha (1-\lambda )}>\frac{ Y_{L}}{Y_{H}}$, and decreases otherwise.

We can sum up the intuitions of the $\phi$-equilibrium and the *s* -equilibrium in the following:

#### Prediction 4

* Tax evasion is not affected if public expenditure provided from the beginning remains low; whereas the effect is ambiguous if public expenditure provided from the beginning is high and in the interest of some taxpayers only, i.e. schooling.*

## Appendix B: Proofs

### Proof of Proposition 1

It is useful to start computing *G*’s expected payoff from each possible strategy.

Suppose *G* sets $\left\{ \tau _{H},e_{H},t\right\}$ in period 1. Only optimistic *T* (proportion $\gamma$) will trust *G* and pay taxes, so *G* has no profitable deviation to lower the tax rate below $\tau _{H}$. In period 2 *G* cannot change $q=t$ but can change the tax rate. However, it does not turn out to be profitable because all *T* will experience quality and would pay taxes if $\tau \le \tau _{H}$. So, if *G* sets $\left\{ \tau _{H},e_{H},t\right\}$ in period 1 it will set $\left\{ \tau _{H},e_{H},t\right\}$ in period 2 as well, getting $(1+\gamma )\mathbf { \tau }_{{\mathbf {H}}}-2c_{T}$.

Suppose *G* sets $\left\{ \tau _{H},e_{H},s\right\}$ in period 1. Only optimistic *T* (proportion $\gamma$) will trust *G* and pay taxes, so *G* has no profitable deviation to lower the tax rate below $\tau _{H}$. In period 2 *G* cannot change $q=s$ and only $T_{S}$ (proportion $\alpha$) and optimistic unaware $T_{T}$ (proportion $(1-\alpha )(1-\lambda )\gamma$) would pay taxes if $\tau =\tau _{H}$. Conversely, all *T* except pessimistic $T_{T}$ who have not experienced quality (proportion $1-(1-\alpha )(1-\lambda )(1-\gamma )$) would pay taxes if $\tau ={\bar{\tau }}$ . In the former case *G* gets $[\gamma +\alpha +(1-\alpha )(1-\lambda )\gamma ]\varvec{\tau }_{{\mathbf {H}}}-2c_{S}$, whereas in the latter *G* would get $\gamma \varvec{\tau }_{{\mathbf {H}}}+[1-(1-\alpha )(1-\lambda )(1-\gamma )]{\bar{\mathbf {\tau }}}-2c_{S}$. It is easy to show that the former strategy dominates the latter because yields a higher payoffit comes from $\varvec{\tau }_{{\mathbf {H}}}>{\bar{\mathbf {\tau }}}$ and $\alpha +(1-\alpha )(1-\lambda )\gamma ]>1-(1-\alpha )(1-\lambda )(1-\gamma )$). So, if *G* sets $\left\{ \tau _{H},e_{H},s\right\}$ in period 1 it will set $\left\{ \tau _{H},e_{H},s\right\}$ in period 2 as well, getting $[\gamma +\alpha +(1-\alpha )(1-\lambda )\gamma ]\varvec{\tau }_{{\mathbf {H}}}-2c_{S}$.

Suppose *G* sets $\left\{ \tau _{H},e_{H},b\right\}$ in period 1. Only optimistic *T* (proportion $\gamma$) will trust *G* and pay taxes, so *G* has no profitable deviation to lower the tax rate below $\tau _{H}$. In period 2 *G* cannot change $q=b$ and only optimistic unaware *T* (proportion $(1-\lambda )\gamma$) would pay taxes if $\tau >\tau _{L}$. Conversely, all *T* would pay if $\tau =\tau _{L}$. In the former case *G* gets $\gamma (2-\lambda )\varvec{\tau }_{{\mathbf {H}}}-2c_{B}$, whereas in the latter *G* would get $\gamma \varvec{\tau }_{{\mathbf {H}}}+\varvec{\tau }_{ {\mathbf {L}}}-2c_{B}$.

Finally, suppose *G* sets $\left\{ {\bar{\tau }},{\bar{e}},\phi \right\}$ in period 1. All *T* will pay taxes, so *G* has no profitable deviation to lower the tax rate. In period 2 *G* is free to keep the same level of expenditure, getting a total payoff of $2({\bar{\mathbf {\tau }}}-{\bar{c}})$, or to increase the level of public expenditure up to $e_{H}$. In the latter case, only optimistic *T* (proportion $\gamma$) would trust *G* and pay taxes if $\tau >\tau _{L}$: the Efficiency Condition proves that *G* strictly prefers $\left\{ {\bar{\tau }},{\bar{e}},\phi \right\}$ over $\left\{ \tau _{L},e_{H},b\right\}$ in period 2, and setting $q\ne b$ is strictly dominated by setting $q=b$ for any $\tau >\tau _{L}$. So, *G* could only set $\left\{ {\bar{\tau }},{\bar{e}},\phi \right\}$ in period 1 and $\left\{ \tau _{H},e_{H},b\right\}$ in period 2, getting ${\bar{\mathbf {\tau }}}-{\bar{c}} +\gamma \varvec{\tau }_{{\mathbf {H}}}-c_{B}$: it is easy to show that this strategy strictly dominates offering $\left\{ \tau _{H},e_{H},b\right\}$ in period 1 and $\left\{ \tau _{L},e_{H},b\right\}$ in period 2 because ${\bar{\mathbf {\tau }}}-{\bar{c}}>\varvec{\tau }_{{\mathbf {L}}}-c_{B}$.

It follows that:

- (*b*-equilibrium) Suppose that *G* sets $\left\{ \tau _{H},e_{H},b\right\}$ in both periods, with optimistic *T* (proportion $\gamma$) paying taxes in period 1 and only optimistic unaware *T* (proportion $(1-\lambda )\gamma$) paying taxes in period 2. The conditions in the premise are necessary to exclude profitable deviations to $\left\{ \tau _{H},e_{H},t\right\}$ or $\left\{ \tau _{H},e_{H},s\right\}$ or $\left\{ {\bar{\tau }},{\bar{e}},\phi \right\}$ in both periods or to $\left\{ {\bar{\tau }},{\bar{e}},\phi \right\}$ in period 1 and $\left\{ \tau _{H},e_{H},b\right\}$ in period 2.
- ($\phi$-equilibrium) Suppose that *G* sets $\left\{ {\bar{\tau }},{\bar{e}} ,\phi \right\}$ in both periods with all *T* paying taxes. The conditions in the premise are necessary to exclude profitable deviations to $\left\{ \tau _{H},e_{H},t\right\}$ or $\left\{ \tau _{H},e_{H},s\right\}$ or $\left\{ \tau _{H},e_{H},b\right\}$ in both periods or to $\left\{ {\bar{\tau }} ,{\bar{e}},\phi \right\}$ in period 1 and $\left\{ \tau _{H},e_{H},b\right\}$ in period 2.
- (*s*-equilibrium) Suppose *G* sets $\left\{ \tau _{H},e_{H},s\right\}$ in both periods, optimistic *T* (proportion $\gamma$) pay taxes in period 1, whereas only $T_{S}$ (proportion $\alpha$) pay taxes in period 2. The conditions in the premise are necessary to exclude profitable deviations to $\left\{ \tau _{H},e_{H},t\right\}$ or $\left\{ \tau _{H},e_{H},b\right\}$ or $\left\{ {\bar{\tau }},{\bar{e}},\phi \right\}$ in both periods or to $\left\{ {\bar{\tau }},{\bar{e}},\phi \right\}$ in period 1 and $\left\{ \tau _{H},e_{H},b\right\}$ in period 2.
- (*t*-equilibrium) Suppose *G* sets $\left\{ \tau _{H},e_{H},t\right\}$ in both periods, only optimistic *T* pay taxes in period 1,  and all *T* pay in period 2. The conditions in the premise are necessary to exclude profitable deviations to $\left\{ \tau _{H},e_{H},s\right\}$ or $\left\{ \tau _{H},e_{H},b\right\}$ or $\left\{ {\bar{\tau }},{\bar{e}},\phi \right\}$ in both periods or to $\left\{ {\bar{\tau }},{\bar{e}},\phi \right\}$ in period 1 and $\left\{ \tau _{H},e_{H},b\right\}$ in period 2. $\square$

## Footnotes

1 Although surveys have the merit of incorporating additional social variables to a structural model, they may turn out to be unreliable or, at least, inaccurate since they are based on self-report. For instance, participants in the survey have to scale their agreement to statements like “how much is justifiable not paying for your ticket on public transport” . Not surprisingly, it may happen that people on average believe that it is not justifiable, but from their answers it is not possible to understand whether they have always paid the ticket for public transportation. On the inaccuracy of surveys see Ellfers et al. (1987).

2 IRAP (Imposta Regionale sulle Attività produttive) is the tax on the net production value at regional level, IRPEF (Imposta Regionale sul Reddito delle Persone Fisiche) is the personal income tax, whereas IRES stands for the corporate income tax.

3 For further details about the whole methodologies that can be applied to evaluate the tax gap, and in particular on how the top-down approach works, see the report on the observed economy and on the tax evasion that is annually published by the Italian Revenue Agency.

4 We do not have observations for the balance sheets of municipalities located in the provinces of Olbia-Tempio, Ogliastra, Medio Campidano and Carbonia Iglesias in Sardinia.

5 Abruzzo, Apulia, Basilicata, Calabria, Campania, Molise, Sardinia and Sicily are considered regions belonging to the south of Italy.

6 Considering the other covariates as endogenous does not change our results.

7 In the Robustness checks section we assess whether results are the same as those presented in the main text of the paper when a difference GMM and a two-step system GMM estimation procedures are implemented.

8 The Stata command is xtgcause. The test works if the panel is strongly balanced and in case both the outcome variable and government size are stationary series (unit-root test results are available upon request).
